
JOURNAL INFORMATION
==============================
NLM Title Abbreviation: JBRA Assist Reprod
Journal ID: JBRA Assist Reprod
NLM Journal ID: 101684552
Journal ID: jbra

JBRA Assisted Reproduction

ISSN: 1517-5693
EISSN: 1518-0557
Publisher: Brazilian Society of Assisted Reproduction

ARTICLE INFORMATION
==============================
PMCID: PMC6724386
PMID: 31436401
DOI: 10.5935/1518-0557.20190051
Article version: 1
Subjects: Oral Presentations

Abstracts of the 23rd Annual Congress of the SBRA, Curitiba/PR, 2019

Publication date: 2019 Jul-Sep
Print publication date: 2019 Jul-Sep
Volume: 23
Issue: 3
First page: 298
Last page: 300
License: This is an Open Access article distributed under the terms of the Creative Commons Attribution License, which permits unrestricted use, distribution, and reproduction in any medium, provided the original work is properly cited.
License URL: https://creativecommons.org/licenses/by/4.0/

==============================


O-01. Psychological Intervention in the Oocyte Pick-up Room and Recovery Room Environments in Assisted Reproduction: new listening accounts

Gusmão M.C.G. 1
Teixeira L.M. 2
Mancebo A.C.A. 1
Souza M.M. de 1
Antunes R.A. 1
Souza M.C.B de 1
1 Fertipraxis - RJ, Brazil
2 Fertipraxis/Hospital Federal da Lagoa - RJ, Brazil

O-02. Priorities for Research in Infertility and Assisted Reproductive Technology Treatments - a James Lind Alliance Priority Setting Partnership with Brazilian Patients

Lorenzon A.R. 1
Garcia D. 2
Silva L. 2
Oliveira C.A. de 3
Chehin M.B. 1
Marinho R.M. 3 4
Caetano J.P.J. 3
Vassena Rita 2
Motta E.L.A. da 1 5
1 Huntington Medicina Reprodutiva, São Paulo, SP - Brazil
2 Clinica Eugin, Barcelona, Spain
3 Pró-Criar Medicina Reprodutiva, Belo Horizonte, MG - Brazil
4 Faculdade Ciências Médicas Minas Gerais, Belo Horizonte, MG, Brazil.
5 Departmento de Ginecologia, Escola Paulista de Medicina, Universidade Federal de São Paulo, São Paulo, SP, Brasil

O-03. Granulocyte colony stimulating factor (G-CSF) role on women submitted to in vitro fertilization associated with thin endometrium: systematic review

Rocha M.N.C. 1
Florêncio R.S. 1
Alves R.R.F. 1
1 Humana Medicina Reprodutiva. Goiânia, GO, Brazil

O-04. Predictive factors for successful pregnancy in an egg-sharing donation program

Braga D.P.A.F. 1 2
Setti A.S. 1 2
Iaconelli Jr. A. 1 2
Borges Jr. E. 1 2
1 Fertility Medical Group- Sao Paulo - SP, Brazil
2 Instituto Sapientiae - Centro de Estudos e Pesquisa em Reprodução Assistida- Sao Paulo - SP, Brazil

O-05. Correlation between morphokinetics parameters and standard morphological evaluation: what can we predict from early embryo development? A time-lapse system experience with 2085 morphologic grading blastocysts

Jacobs C. 1
Nicolielo M. 1
Erberelli R. 1
Mendez F. 1
Fanelli M. 1
Cremonesi L. 1
Aiello B. 1
Lorenzon A.R. 1
1 Huntington Medicina Reprodutiva, São Paulo, SP, Brazil

O-06. Cancer fertility preservation: a report from a Brazilian social program

Berton C. 1
Brogliato C. 1
Yoshida I. 1
Vellez L. 1
Suganuma C. 1
Barbosa C. 1
Cordts E. 1
1 Instituto Idéia Fértil de Saúde Reprodutiva, Santo André/SP, Brasil

O-07. Expansion and herniation: evaluation of the best predictor related to pregnancy rate after quarter laser assisted hatching in frozen blastocysts transfer

Brogliato C. 1
Romanini J. 1
Berton C. 1
Suganuma C. 1
Vellez L. 1
Yoshida I. 1
Barbosa C. 1
1 Instituto Idéia Fértil de Saúde Reprodutiva, Santo André/SP, Brazil

O-08. ICSI in late matured oocytes, is it worth it? Study with laboratory, clinical and genetic evaluation results

Vellez L. 1
Brogliato C. 1
Berton C. 1
Yoshida I. 1
Barbosa C. 1
Cordts E. 1
1 Instituto Idéia Fértil de Saúde Reprodutiva, Santo André/SP, Brasil

